# Plasma Lipid Profiling Identifies Biomarkers of Cerebral Microvascular Disease

**DOI:** 10.3389/fneur.2019.00950

**Published:** 2019-08-29

**Authors:** Ida Azizkhanian, Sunil A. Sheth, Anthony T. Iavarone, Songmi Lee, Visesha Kakarla, Jason D. Hinman

**Affiliations:** ^1^New York Medical College School of Medicine, Valhalla, NY, United States; ^2^Department of Neurology, UT Health McGovern School of Medicine, Houston, TX, United States; ^3^QB3/Chemistry Mass Spectrometry Facility, University of California, Berkeley, Berkeley, CA, United States; ^4^Department of Neurology, David Geffen School of Medicine, University of California, Los Angeles, Los Angeles, CA, United States

**Keywords:** sphingolipids, stroke, white matter (WM), biomarker, ceramide (CER), sphingomyelin (SM)

## Abstract

Brain-specific sphingolipids (SLs) may serve as effective biomarkers of white matter hyperintensities (WMH). Here, we investigate the efficacy of SLs as a novel fluid-based biomarker to identify WMH reflective of chronic ischemia. Patients presenting to our stroke center for evaluation of acute neurological deficits were enrolled in the Advanced Serum Profiling in Recent Stroke (ASPIRE) study. From this cohort of 202 individuals, 58 patients who underwent an MRI and did not have a clinical stroke event were included in this study. Plasma samples were collected at the time of MRI, and targeted SL profiling was performed by HPLC/tandem mass spectrometry. T_2_ FLAIR imaging was evaluated for WMH and scored according to the Fazekas scoring (FS) method and manually quantified. Twenty two SLs were definitively identified, consisting of ceramide (Cer) and sphingomyelin (SM) species. Of these, two sphingolipids, SM 38:1 and Cer 34:1, significantly correlated with high FS (*r* = 0.287, *p* = 0.029, and *r* = 0.356, *p* = 0.006, respectively) and were used in subsequent analysis. SM 38:1 (OR 1.129, 95% CI 1.032, 1.236, *p* = 0.008) and Cer 34:1 (OR 1.118, 95% CI 1.031, 1.212, *p* = 0.007), accurately differentiated between FS 0–2 vs. 2.5–6 in regression analysis. A combined lipid score demonstrated fair discrimination in ROC analysis (AUC = 0.729, *p* = 0.003) and was cross-validated using leave-one-out analysis. Plasma levels of brain-specific SLs may serve as effective biomarkers of subacute white matter disease.

## Introduction

White matter hyperintensities (WMH) detected on T_2_ weighted images are associated with white matter damage and can be identified in 11% of adults over 60 and in 94% of adults over 82 (1). While age is the most consistent risk factor for WMH; diabetes, smoking, and hypertension are also independent risk factors, suggesting that WMHs are pathological rather than a part of the normal aging process (2). A meta-analysis of 22 imaging studies evaluating WMH shows that these lesions are associated with increased risk of stroke (hazard ratio 3.3) and dementia (hazard ratio 1.9) (3). Increased WMH load is also associated with increased severity of cognitive dysfunction (4). WMH are associated with poor recovery after stroke and hemorrhagic transformation after thrombolysis (3, 5–9). Other evidence supports an association with gait disturbances and impaired balance, putting those with significant WMH load at risk for falls and hospitalizations (2). Populations with limited access to healthcare and MRI studies may have underreported WMH and unrecognized cognitive impairment mandating alternative detection methods for WMH in these populations to promote screening, early intervention and appropriate management to prevent complications and hospitalizations.

Existing blood biomarkers for cerebral vascular disease are not specific to WMH and are not reliably detected in subclinical, or early stages of disease (10). While BNP and CRP are elevated in acute stroke and have shown modest ability to predict risk of future stroke, these biomarkers fail to reflect current extent of cerebral injury (11, 12). Non-specific inflammatory markers such as CRP, TNF, and IL-6, have not been shown to relate to WMH load (13). While some markers of endothelial damage, such as von Willebrand factor and intercellular adhesion molecule 1 are not associated with increasing WMH volume, others, including circulating vascular adhesion molecule-1, are (13, 14). Therefore, the role of different inflammatory pathways in the progression of WMH remains unclear. Serum neurofilament light chain (sNfL) has been recently shown to be a brain-specific biomarker for acute ischemic stroke (15, 16). While sNfL levels are reported to increase with acute ischemic damage and WMH, levels also increase in other neurological disorders including motor neuron disease (MND), multiple sclerosis (MS), and Alzheimer's disease (AD) (17–20). Because AD, MND, and WMH are all more prevalent in the older patient population, sNfL can only be used as a non-specific biomarker for WMH.

Tissue pathology from regions of WMH reveals demyelination, axonal damage, and mild gliosis (21), arising from chronic microvascular dysfunction. Oligodendrocyte vacuolization around venules in perivascular spaces contributes to loss of myelination, axonal damage and white matter fiber disruption in regions of WMH. Both histological and diffusion tensor imaging evidence suggest that white matter changes extend into a penumbra surrounding the region of WMH detected by routine MRI (22–24). Therefore, MRI may underestimate extent of brain injury and can fail to detect early changes. Plasma biomarkers that reflect direct damage to white matter may serve a critical role in identifying early white matter injury.

We previously demonstrated that sphingolipids (SLs) are highly enriched in the brain and that plasma SLs are sensitive markers of acute ischemic cerebral injury. SLs, and in particular one subset of SLs known as sphingomyelins, are known to be key components of cerebral white matter, as major constituents of the cerebral myelin sheaths that contribute to membrane fluidity and structural integrity (25). In this study, we determine the ability of circulating SLs to serve as accurate biomarkers of WMH.

## Subject/Materials and Methods

### Ethics Statement

Research involving human subjects was approved by the Institutional Review Board of the University of California, Los Angeles (IRB # 14-001798) and was conducted in compliance with the Health Information Portability and Accountability Act. A board-certified Neurologist interviewed all patients to determine the capacity of each individual participant to provide informed consent. If in the opinion of the physician the patient suffered from a compromised ability to provide consent, a surrogate consent procedure was instituted whereby the next of kin or legally authorized representative was given the opportunity to consent on the patient's behalf. Formal written consent was obtained for all participants prior to the collection of blood samples.

### Study Design

This cohort study was prospectively designed to build from the results of our previous discovery-phase study, to validate findings on lipid biomarkers of acute ischemic stroke in a larger, real-world clinical cohort. Consecutive participants were patients presenting to the Ronald Reagan UCLA Emergency Department with symptoms concerning for stroke between December 2014 and June 2016 and who were offered the opportunity to participate in the study. Patients were included in this study if they had onset of stroke symptoms within 8 h of presentation (or within 2 h of presentation if symptoms were present upon awakening from sleep and the time of symptom onset was unknown); were >18 years of age and were able to offer informed consent or had a suitable surrogate individual who could consent on their behalf. The final clinical diagnosis was based on the results of a detailed evaluation of each patient by the Neurovascular Neurology service. Blood samples were collected by peripheral vein venipuncture into heparin-containing tubes. Samples were kept on ice and then centrifuged immediately at 13,000x g for 5 min at 4°C. The plasma was collected and aliquoted into freezer vials for storage at −80°C. Subjects with evidence of CNS infection, known CNS malignancy, or recent head trauma as a potential cause of neurologic symptoms were excluded. There were no other neurological conditions that met criteria for exclusion. In this pre-specified *post hoc* subset analysis, patients were included if they underwent MRI at the time of blood collection, and were not ultimately diagnosed as having an acute ischemic stroke or TIA. Descriptive statistics are presented on the cohort as a whole, and then for the purposes of model derivation to identify potential biomarkers of subacute cerebral microvascular disease, only stroke mimic cases were used and a leave-one-out validation was performed. Patients with stroke mimics had a range of pathologies including migraines, brain tumors, and syncope (Table 1).

**Table 1:** Stroke mimic pathologies.

**Stroke mimic**	**Number (%)**
Migraine	12 (20.7)
Seizure	7 (12)
Anxiety	3 (5.2)
Bell's palsy	2 (3.4)
Dementia	2 (3.4)
Tumor	2 (3.4)
Hypertension	4 (6.9)
Acute coronary syndrome	2 (3.4)
Syncope	2 (3.4)
Vestibular	3 (5.2)
Intoxication	1 (1.7)
Multiple sclerosis	1 (1.7)
Other/unknown	6 (10.3)

### Sphingolipid Extraction

Lipid extraction and detection were performed in an identical manner as previously reported (26). Briefly, 20 μL of plasma was placed in 13 × 100 mm screw-capped borosilicate glass test tubes with Teflon caps (Fisher Scientific Catalog Number 14-933A, New Jersey, USA). 0.5 mL of methanol followed by 0.25 mL of chloroform (Fisher Scientific, New Jersey, USA) and 10 μL of internal standards (Sph/Cer Mixture I, Catalog Number LM-6002, Avanti Polar Lipids, Alabaster, AL) were added. Samples were then sonicated in an ultrasonic bath until they appeared evenly dispersed and then incubated for 2 h at 48°C in a heating block. Tubes were then cooled to room temperature and 75 μL of 1M KOH was added. This mixture was sonicated briefly and then incubated for 30 min at 37°C. Samples were then cooled to room temperature and neutralized by addition of 16 μL of glacial acetic acid (Fisher Scientific, New Jersey, USA). pH was checked with test strips to verify near return to neutral pH 7.0. One milliliter of chloroform and 2 mL of water were added to each tube. The solution was mixed gently and centrifuged at 300 x g for 5 min to separate the phases. A Pasteur pipette was rinsed with chloroform and then used to remove the lower layer into another glass test tube, and the solvent was removed using vacuum centrifugation. The lipid residue was then re-dissolved in 75 μL methanol. The upper phase was re-extracted by adding 1 mL chloroform, mixing gently, and centrifuging as above. The lower layer was again transferred to another glass test tube using a Pasteur pipette that had been rinsed with chloroform, and the solvent was removed using vacuum centrifugation. The lipid residue was re-dissolved in 75 μL methanol. These two extracts were then pooled for a total of 150 μL, vortexed, and then centrifuged at 13,000 x g to clarify. The supernatant was then transferred to an autosampler vial (Catalog number 225180, Wheaton, New Jersey, USA). Vials were stored at −80°C until ready for LC-MS analysis.

### High Performance Liquid Chromatography-Mass Spectrometry

Methanol (Optima grade, Fisher Scientific, New Jersey, USA), formic acid (99+%, Thermo Fisher Scientific, Waltham, MA), ammonium formate (99%, Alfa Aesar, Ward Hill, MA), and water purified to a resistivity of 18.2 MΩ·cm (at 25°C) using a Milli-Q Gradient ultrapure water purification system (Millipore, Billerica, MA), were used to prepare mobile phase solvents for liquid chromatography-mass spectrometry. Lipid extracts were analyzed using an Agilent 1200 liquid chromatography system (Agilent, Santa Clara, CA) that was connected in-line with an LTQ Orbitrap XL mass spectrometer equipped with an electrospray ionization source (ESI; Thermo Fisher Scientific, Waltham, MA). The LC was equipped with a C4 analytical column (Viva C4, length: 150 mm, inner diameter: 1.0 mm, particle size: 5 μm, pore size: 300 Å, Restek, Bellefonte, PA), and a 100-μL sample loop. Solvent A was 99.8% water/0.2% formic acid and solvent B was 99.8% methanol/0.2% formic acid (volume/volume). Solvents A and B both contained 5 mM ammonium formate. Lipid extract samples in autosampler vials sealed with septa caps were loaded into the autosampler compartment prior to analysis. The sample injection volume was 100 μL. The elution program consisted of isocratic flow at 30% B for 3 min, a linear gradient to 50% B over 0.5 min, a linear gradient to 100% B over 11.5 min, isocratic flow at 100% B for 5 min, a linear gradient to 30% B over 0.5 min, and isocratic flow at 30% B for 19.5 min, at a flow rate of 150 μL per min. The column and sample compartments were maintained at 40 and 4°C, respectively. The injection needle was rinsed with a 1:1 methanol:water (volume/volume) solution after each injection to avoid cross-contamination between samples.

The column exit was connected to the ESI probe of the mass spectrometer using PEEK tubing (0.005″ inner diameter × 1/16″ outer diameter, Agilent, Santa Clara, CA). Mass spectra were acquired in the positive ion mode over the range *m/z* = 270–1,150 using the Orbitrap mass analyzer, in profile format, with a mass resolution setting of 100,000 (at *m/z* = 400, measured at full width at half-maximum peak height). In the data-dependent mode, the five most intense ions exceeding an intensity threshold of 30,000 counts were selected from each full-scan mass spectrum for tandem mass spectrometry (MS/MS) analysis using collision-induced dissociation (CID) or pulsed-Q dissociation (PQD). MS/MS spectra were acquired in the positive ion mode using the linear ion trap, in centroid format, with the following parameters: isolation width 3 *m/z* units, normalized collision energy 30%, activation time 30 ms, activation Q 0.25 for CID, activation Q 0.50 for PQD, and default charge state 1+. A parent mass list was used to preferentially select ions of interest for MS/MS analysis. To avoid the occurrence of redundant MS/MS measurements, real-time dynamic exclusion was enabled to preclude re-selection of previously analyzed precursor ions, using the following parameters: repeat count 2, repeat duration 30 s, exclusion list size 500, exclusion duration 60 s, and exclusion width 0.1 *m/z* unit.

### Sphingolipid Identification

Raw Thermo MS data files were converted into centroided data and into the mzXML format using the MS Convert tool in the ProteoWizard 3.0 package (27). Retention time correction and automated peak picking were then performed using XCMS online (28). Feature detection was performed using the centWave method at 2 parts per million (ppm) *m/z* deviation, minimum peak width of 20 s and maximum peak width of 80 s. A 20 s retention time deviation (bw) was used with a width of 0.01 s for overlapping *m/z* slices (mzwid). Peaks were annotated by searching for isotopes plus selected adducts (H^+^ and Na^+^) with an *m/z* absolute error of 0.015 and relative error of 5 ppm. Parameters were iteratively optimized by manual review of the selective peaks.

From the automatically selected peaks, target lipids were identified from the METLIN database with the annotation parameters described above (Scripps Research Institute, La Jolla, CA). Peaks were then filtered to only include SLs, and then manually reviewed to ensure accuracy. Peaks that were felt to be inappropriately selected were excluded. Thus, the final list of included SLs consisted of automatically selected peaks that were manually curated.

### Imaging Analysis

MRI was performed on either Siemens Avanto 1.5T or Siemens Trio 3T machines. Axial T_2_-weighted images were obtained continuously in 5-mm-thick sections with repetition time of 3,800 ms and time to echo of 116 ms. The field of view was 220 cm, and the matrix was 384 × 384. Axial FLAIR images were obtained continuously in 5-mm-thick sections with repetition time of 9,000 ms and time to echo of 89 ms. The field of view was 220 cm, and the matrix was 320 × 216. Axial diffusion-weighted images were obtained continuously in 5-mm-thick sections with repetition time of 5,600 ms and time to echo of 106 ms. The field of view was 255 cm, and the matrix was 192 × 192.

Two authors (I.A. and V.K.), who were blinded to clinical data, evaluated WMH on axial T_2_-weighted FLAIR images. The raters used the Fazekas subjective scoring method to assign a score from 0 to 3 in periventricular white matter (PVWM) and deep white mater (DWM) regions of the brain (29, 30). PVWM hyperintensities were scored 0, no hyperintensities; 1, caps or pencil thin lining; 2, smooth halo; 3, irregular periventricular signal extending into the DWM. The DWM was scored 0, no hyperintensities; 1, punctate foci; 2, beginning confluence; 3, large confluent areas. The total Fazekas score (FS) was obtained by summing the scores from periventricular and deep white matter regions. Interrater reliability as measured by quadratic weighted Cohen's Kappa was excellent with κ = 0.81. The average of the scores assigned by the two authors was used in subsequent analysis. This endpoint was dichotomized to separate low WMH load (FS 0–2) from high WMH load (FS > 2). Other features of cerebral microvascular disease including microbleeds and perivascular spaces were also evaluated using axial gradient-echo sequences and axial T_2_-weighted sequences.

### White Matter Hyperintensity Segmentation

WMH area was quantified using manual ROI segmentation in OsiriX Lite (Pixmeo SARL). WMH areas on individual T_2_-Weighted FLAIR MRI slices were carefully outlined using the wand tool and the total area of each lesion measured. Lesion areas were summed across slices, and classified as deep or periventricular. Deep and periventricular areas were calculated and then correlated with Fazekas scores and lipid species.

### Statistical Analysis

Statistical analysis was performed using SPSS, Graphpad v 7.0, and Matlab v2018a software. Continuous variables with a normal distribution were described as mean ± SD, and non-normally distributed variables were described as median and interquartile range. For univariate analysis, categorical variables were compared by χ^2^ statistics. A Spearman's correlation was used to measure the association between lipid species, FS, and measured WMH areas. Logistic regression analyses were performed to distinguish independent predictors of heavy white matter damage defined as previously described FS cut-offs. Leave-one-out cross-validation was performed in Matlab using defined code. Statistical significance was defined as *p* < 0.05.

## Results

### Subjects

Demographic, clinical, radiographic, and plasma specimens were collected as part of the Advanced Serum Profiling in Recent (ASPIRE) Stroke study, a previously conducted UCLA IRB approved, single-center observational study that aimed to identify lipid biomarkers of acute stroke. Data for the ASPIRE trial were collected from all consenting patients over the age of 18 years old presenting to the emergency department (ED) between December 2014 and June 2015 with acute neurological symptoms within the prior 24 h. Subjects with evidence of CNS infection, known CNS malignancy, or recent head trauma as a potential cause of neurologic symptoms were excluded. Demographic data and neurodiagnostic studies are available for all enrolled patients.

**Figure 1:**
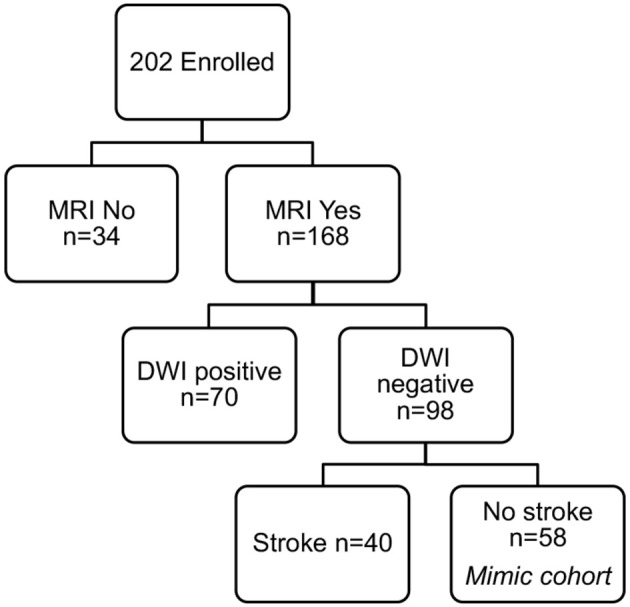
ASPIRE Study subcohorts. The total ASPIRE Study Cohort of enrolled subjects was segmented into those with and without MRI to facilitate accurate measurement of white matter WMH burden. Individual subjects with DWI-positive scan indicating acute ischemia were excluded. Subjects with DWI-negative scans were further subdivided based on clinical diagnosis of stroke. The remaining 58 subjects presented with a neurologic mimic of stroke, did not have neuroimaging evidence of acute pathology, and were included in the analysis for plasma sphingolipid biomarkers.

**Figure 2:**
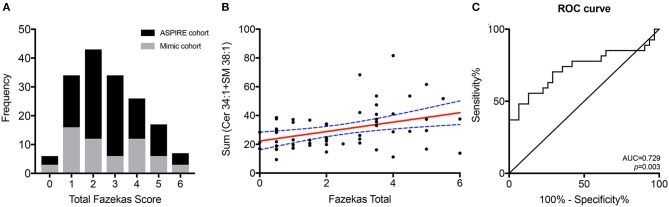
Combined lipid score predicts WMH. The distribution of Fazekas scoring for the total ASPIRE study cohort (black) and the stroke mimic cohort (gray) **(A)**. Correlation of the combined Cer 34:1 + SM 38:1 lipid score with Fazekas scoring in the stroke mimic cohort **(B)**. ROC curve for the combined lipid score as a predictor of WMH **(C)**.

To identify only those subjects with subacute white matter disease, the total study population was selectively reduced to exclude those presenting with imaging or clinically confirmed TIA/stroke and those without MRI imaging. Of the 202 patients enrolled in the ASPIRE Stroke study, 34 were excluded as they had not undergone MRI, and an additional 110 were excluded for being diagnosed ultimately with acute ischemic stroke or TIA (Figure 1). Among the 58 patients who presented with a stroke mimic syndrome and were included in this analysis, median age was 69.5 and 51.7% were female (Table 2). Higher FS was associated with advanced age (29 vs. 70% over 70 yo, FS 0–2 vs. 2.5–6, *p* = 0.002). While sex did not have an effect on FS in χ^2^ analysis (*p* = 0.32), age did (*p* = 0.0041). Fazekas positive status was given to 46.5% of the cohort for having FS > 2. Rates of other stigmata of cerebral microvascular disease in the stroke mimic cohort were low with only 5.5% having cerebral microbleeds and 17.8% having prominent perivascular spaces, the majority (>70%) of which also have FS > 2. Figure 2A demonstrates the distribution of Fazekas scores in the total ASPIRE cohort and the smaller stroke mimic cohort. Stroke mimics represented a variety of non-ischemic diagnoses ranging from migraine to syncope (Table 1).

**Table 2:** Demographics.

**ASPIRE cohort**	**Total**	**FS–**	**FS+**	**FS+** **vs. –**	
	***n*** **(%)**			**χ^**2**^**	***p***
Total	168 (100)	83 (49.4)	85 (50.6)	0.048	0.83
Male	92 (54.8)	45 (54.2)	47 (55.3)	0.02	0.89
Female	76 (45.2)	38 (45.8)	38 (44.7)	0.02	0.89
MetS	42 (25)	21 (25)	21 (25)	0	1
Hx statin	63 (37.5)	17 (20)	43 (51.8)	18	<0.0001^**^
Hx stroke	63 (39.9)	43 (50.7)	18 (21.7)	15.2	0.0001^**^
	**μ** **(SD)**			***t***	***p***
Age	70.4 (15.2)	62.6 (15.5)	78.4 (9.9)	7.89	<0.0001^**^
SBP	158 (30.8)	154.4 (27.8)	162 (33.3)	1.6	0.11
DBP	85.5 (17.2)	86.9 (16.5)	84.2 (18.1)	−1.01	0.31
Glucose	133.8 (62.1)	138.5 (76)	129 (44.1)	−0.994	0.32
**Stroke mimic cohort**	**Total**	**FS**–	**FS+**	**FS+** **vs. –**	
	***n*** **(%)**			***χ***^**2**^	***p***
Total	58 (100)	31 (53.4)	27 (46.5)	0.548	0.46
Male	28 (48)	19 (61.3)	9 (33.3)	4.45	0.035^*^
Female	30 (51.7)	12 (38.7)	18 (66.7)	4.45	0.035^*^
MetS	11 (19)	4 (12.9)	7 (25.9)	1.56	0.21
Hx statin	23 (39.7)	8 (25.8)	15 (55.6)	5.26	0.022^*^
Hx stroke	25 (43.1)	7 (22.6)	18 (66.7)	11.25	0.0008^**^
	**μ** **(SD)**			***t***	***p***
Age	66.12 (17.5)	59 (18.1)	74 (9)	3.9	0.0003^**^
SBP	154 (30.7)	144.3 (26)	164.6 (32)	2.67	0.01^**^
DBP	87.2 (18.7)	86.3 (17.8)	88.1 (19.9)	0.36	0.72
Glucose	123.3 (48.6)	120.6 (55.6)	126.2 (40.7)	0.43	0.67

*n (%), number (percent); p, level of significance; MetS, Metabolic Syndrome; χ^2^, chi squared statistic; Hx Statin use, History of statin use; Hx Stroke, History of Stroke; Fazekas –, Fazekas score ≤ 2; Fazekas +, Fazekas score >2; $\bar{x}$ Age (SD), mean age (standard deviation); MSBP (IQR), median systolic blood pressure (interquartile range); M DBP (IQR), median diastolic blood pressure (interquartile range)*.

** *Correlation is significant at the 0.01 level (2-tailed)*.

* *Correlation is significant at the 0.05 level (2-tailed)*.

### Sphingolipid Analysis

Two species, SM 38:1 and Cer 34:1, significantly correlated with FS in a Spearman's correlation model in both the overall ASPIRE cohort and the stroke mimic subgroup (Tables 3, 4). In multivariate logistic regression, SM 38:1 (OR 1.129, 95% CI 1.032, 1.236, *p* = 0.008) and Cer 34:1 (OR 1.118, 95% CI 1.031, 1.212, *p* = 0.007) also accurately differentiated between FS 0–2 vs. 2.5–6. When controlling for age, the two species were still independently predictive of WMH load: SM 38:1 (OR 1.125, 95% CI 1.010, 1.253, *p* = 0.032) and Cer 34:1 (OR 1.096, 95% CI, 1.002, 1.200, *p* = 0.046).

**Table 3:** Lipid screening analysis in stroke mimic cohort.

**Lipids**	**M [IQR]**		**Spearman correlation**	
	**Total**	**FS+**	***r***	***p***
SM 34:1	3809.5 [3148.2]	3975 [2793.2]	0.132	(*p* = 0.324)
SM 36:1	1343.7 [803.7]	458.6 [918.2]	0.182	(*p* = 0171)
SM 40:2	1021.3 [439.6]	1015.5 [403.6]	0.032	(*p* = 0.813)
SM 34:2	779.6 [609.2]	793.1 [643.4]	0.078	(*p* = 0.559)
SM 42:1	873.4 [409.1]	869 [396]	0.039	(*p* = 0.769)
SM 38:1	691.6 [402.0]	702.7 [317.5]	−0.035	(*p* = 0.792)
SM 36:2	632.0 [316.9]	667 [357.4]	0.173	(*p* = 0.193)
SG 19:3	492.6 [307.2]	452.3 [322.7]	−0.121	(*p* = 0.364)
SM 38:2	378.7 [174.6]	406.9 [175.7]	0.151	(*p* = 0.258)
SG 20:1	237.2 [189.5]	225 [219.7]	−0.112	(*p* = 0.403)
SG 18:1	242.7 [183.6]	237.3 [231.6]	−0.107	(*p* = 0.426)
SM 39:1	190.1 [134]	181.8 [117.2]	−0.012	(*p* = 0.93)
SM 35:1	177.4 [91.4]	193.9 [97.4]	0.291	(*p* = 0.027)^*^
SM 40:3	143.4 [67.9]	156.3 [65.8]	0.194	(*p* = 0.145)
SM 37:1	95.4 [45.7]	105.9 [53.2]	0.215	(*p* = 0.105)
SM 43:2	86.0 [44]	97.0 [46.4]	0.270	(*p* = 0.04)^*^
SM 43:1	75.6 [46.2]	78.4 [38.8]	0.169	(*p* = 0.204)
SM 39:2	42.8 [19]	45.2 [22.7]	0.186	(*p* = 0.163)
SM 36:3	35.1 [32.8]	38.6 [37.0]	0.167	(*p* = 0.209)
SM 38:3	23.7 [11.8]	26.2 [14.6]	0.235	(*p* = 0.076)
SM 38:1	16.9 [9.4]	20.3 [12.3]	0.287	(*p* = 0.029)^*^
CR 34:1	13.3 [11.7]	16.5 [14.5]	0.356	(*p* = 0.006)^**^

*p, level of significance; r, Spearman's rho; Fazekas +, Fazekas score >2; M (IQR), median (interquartile range)*.

** *Correlation is significant at the 0.01 level (2-tailed)*.

* *Correlation is significant at the 0.05 level (2-tailed)*.

**Table 4:** Lipid screening analysis in ASPIRE cohort.

**Lipids**	**Spearman correlation**		**Linear regression**		
	***r***	***p***	**β**	***p***	**95% CI β**
SM 34:1	0.032	0.728			
SM 36:1	0.061	0.513			
SM 40:2	−0.091	0.323			
SM 34:2	−0.016	0.860			
SM 42:1	−0.090	0.330			
SM 38:1	−0.094	0.310			
SM 36:2	0.028	−0.143			
SG 19:3	−0.143	0.121			
SM 38:2	−0.051	0.585			
SG 20:1	−0.140	0.880			
SG 18:1	−0.019	0.842			
SM 39:1	−0.137	0.137			
SM 35:1	0.099	0.285			
SM 40:3	0.149	0.105			
SM 37:1	0.051	0.578			
SM 43:2	0.097	0.296			
SM 43:1	−0.055	0.554			
SM 39:2	0.066	0.474			
SM 36:3	0.067	0.468			
SM 38:3	0.152	0.100			
SM 38:1	0.275	0.002^**^	0.051	0.010	(1.167, 2.472)
CR 34:1	0.375	0.001^**^	0.062	<0.001	(0.032, 0.093)

*p, level of significance; r, Spearman's rho; 95% CI, 95% confidence interval*.

** *Correlation is significant at the 0.01 level (2-tailed)*.

**Figure 3:**
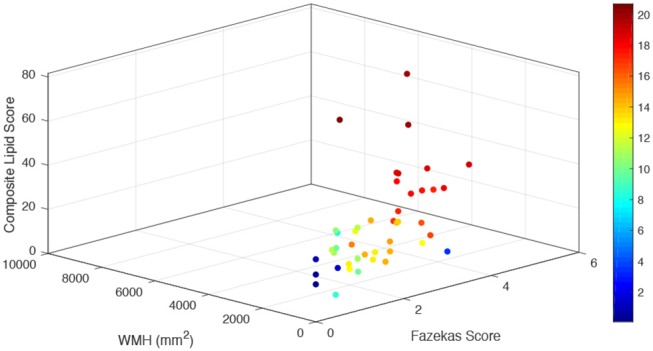
Relationship of combined LS with subjective and objective white matter hyperintensity measurements. For each stroke mimic cohort subject, combined lipid score values are plotted on the vertical z axis (arbitrary units) as a function of measured white matter hyperintensities (mm^2^) (left bottom axis) and Fazekas score (right bottom axis). Color scale corresponds to the summated log-normalized composite of each input variable (LS, WMH area, and FS).

A composite lipid score (LS) was computed from the sum of these two lipid species and demonstrated fair discrimination in ROC analysis (Figures 2B,C). Leave-one-out validation demonstrated a cross-validated *R*^2^ of 12.54%, indicating reproducibility of the LS to predict WMHs resulting from cerebral microvascular disease. To further validate the specificity of the LS, we measured WMH area using FLAIR sequences within the stroke mimic cohort of ASPIRE. Of the 58 stroke mimic subjects, 44 subjects had scans allowing precise measurement of WMH. WMH burden correlated with FS (*r* = 0.828; *p* = 4.1 × 10^−12^) and significantly correlated with LS (*r* = 0.459; *p* = 0.0017). Figure 3 demonstrates the relationship between FS, LS, and WMH in this cohort. In linear regression analysis Cer 34:1 significantly associated with objective white matter measurements (*p* = 0.003), even when controlling for age as a covariate (*p* = 0.003). To evaluate other potential variables to include in the model, mean LS was compared in patients with and without hypertension, diabetes, history of statin use, sex, and low HDL levels. No significant differences in mean LS exist between these groups. Furthermore, these variables were not predictive of FS+ status in univariate binary logistic regression and were therefore excluded from the model.

To demonstrate the specificity of LS for clinically subacute WMH and not the presence of acute ischemic damage, SM 38:1, Cer 34:1, and LS levels were compared between the stroke mimic cohort (*n* = 58) and DWI positive stroke group (*n* = 110). While LS correlated with FS in acute stroke patients (*r* = 0.440, *p* < 0.001), overall SM 38:1 and LS levels were significantly lower in the acute stroke patients compared to stroke mimics (Table 5). There was a trend showing lower Cer 34:1 in the acute stroke patients as well, however, this difference did not reach statistical significance.

**Table 5:** Lipid biomarker levels in stroke mimic and tissue-defined stroke cohorts.

	**Stroke mimic cohort *n* = 58**	**Tissue-defined stroke cohort *n* = 110**	
	**M**		***p***
SM 38:1	15.5	12.8	0.0059^**^
Cer 34:1	11.68	9.67	0.053
LS	28.27	22.46	0.011^**^

*Medians were compared with a one tailed Mann Whitney U-test*.

** *, significance at the 0.01 level. LS, combined lipid score from sum of SM 38:1 and Cer 34:1; M, median; p, p-value*.

## Discussion

Biomarkers play an important role in timely detection and management of neurological diseases. The availability of a fluid-based biomarker for subacute ischemic white matter injury may aid in early detection of subacute ischemic brain damage and prevention of its progression to symptomatic stroke, global cognitive decline, and functional loss. Currently, no brain-specific biomarkers are in clinical use for the evaluation of WMH. WMH are a neuroimaging finding that may be indicative of ischemic brain damage and are often found incidentally yet convey considerable cerebrovascular risk. The high cost and relative inaccessibility of MRI studies for subacute neurological disease necessitate an alternative to neuroimaging for evaluation of chronic white matter disease.

A number of studies have suggested protein-based circulating biomarkers for ischemic white matter injury but these associations are highly variable among populations (31). Recent work suggests that serum detection of neurofilament light chain is transiently increased within 3 months of a recent small subcortical infarct, adding sensitivity to MRI confirmation of acute stroke but not necessarily reliable in the detection of subacute ischemic white matter injury (32). Here, we demonstrate that serum levels of two brain-specific lipid species, SM 38:1 and Cer 34:1, correlate with WMH load as measured by FS specifically in subjects with imaging confirmation of no recent brain ischemia. Summing the relative intensities in serum of these lipids yields a LS that reliably distinguishes between low and high WMH load with a cut off of FS >2. Quantitative assessments of WMH area further validated the role of LS as a blood-based biomarker for WMH in a clinically relevant study population.

An ideal biomarker for cerebrovascular disease is specific to the brain and can cross the blood-brain barrier (BBB) to be measured in serum or plasma from peripheral circulation. Leakage across the BBB is increasingly recognized as central to the pathogenesis of cerebral microvascular disease with evidence of fibrinogen and other plasma proteins leaking into the brain and white matter in individuals with cerebral microvascular disease (32, 33). While acute stroke can cause adequate disruption to the BBB for lipid species to cross over into systemic circulation, other conditions such as acute infection, migraines, or seizures may cause similar leaks and lead to false positive signals. However, subclinical ischemia is generally confined to the cerebral white matter and using a biomarker specific to myelin components may help tune specificity to white matter damage. Indeed, characterization of specific sphingolipid species indicates that those species increased in acute stroke (Cer 42:1 and SM 36:0) are independent and distinguishable from those relevant to WMHs (Cer 34:1 and SM 38:1) (26).

Brain white matter is enriched for the lipid species investigated in this study. Composition of human brain white matter includes sphingomyelin and ceramide (34). Human myelin within white matter tracts comprises many different sphingomyelin species (26, 35). Both ceramide and sphingomyelin are key components of lipid rafts that play a key role in myelination and the maintenance of myelin (36, 37). CSF measurement of sphingomyelin species has been proposed as a biomarker for demyelination in peripheral neuropathies (38). Given the relative abundance of myelin lipids to axonal protein components such as NF-L, leakage of these lipids into the periphery after injury may be a more sensitive indicator of subacute white matter injury. The design of this study enriches for chronic WMH but limits the ability to judge the specificity of LS in other demyelinating injuries.

The original ASPIRE Stroke study cohort of over 200 patients was initially reduced to only 58 to stringently select for those without acute stroke or TIA yet with evidence of WMH reflective of chronic ischemic damage. Despite excluding patients with acute stroke, this cohort is enriched for systemic disease and advanced age due to the original recruitment criteria of the ASPIRE study. Moreover, our approach of using routine clinical imaging as opposed to research grade MRI sequencing approaches that are often used in evaluation of white matter hyperintensity burden, necessarily reduces the accuracy of white matter hyperintensity load measurements but increases the potential applicability of the LS biomarker in clinical practice. Additional studies using precise volumetric measurements of WMH should be pursued to further validate this biomarker. Details on the microstructural integrity of white matter available using DTI images may also enhance the biological significance of sphingolipids through indirect measurement of myelin sheath breakdown. In ASPIRE, rates of other imaging metrics of cerebral microvascular disease including cerebral microbleeds and perivascular spaces were low or potentially confounded by recent ischemia in the stroke cohort and therefore not included in this analysis. However, since SLs are a major constituent of the myelin sheath, WMH are the logical imaging variable to utilize in determining the value of a fluid-based biomarker for cerebral microvascular disease. Further investigation of this biomarker in healthier and more diverse populations and those with other neurologic and white matter injury phenotypes will help to establish baseline values in both healthy and diseased patients. Furthermore, following patients over months and years will establish the longitudinal value of plasma sphingolipid levels to track longitudinal progression of WMH and clinical outcomes associated with WMH such as stroke risk, cognitive decline, gait changes, and other neuropsychiatric variables.

A fluid-based biomarker for cerebral microvascular disease can serve as a valuable tool for detecting subacute brain ischemia. Exposing subclinical ischemia before its progression to symptomatic stroke or clinically apparent cognitive impairment can reduce disease burden in elderly and frail individuals already at elevated risk for acute stroke. Future studies will likely expand the role of this and other fluid-based biomarkers for WMH and provide a critical role for risk stratification in the study of stroke and vascular cognitive impairment and dementia.

## Data Availability

The datasets generated for this study are available on request to the corresponding author.

## Author Contributions

IA, JH, and SS: conception and design of the study and drafting a significant portion of the manuscript or figures. IA, JH, SS, AI, SL, and VK: acquisition and analysis of data.

### Conflict of Interest Statement

The authors declare that the research was conducted in the absence of any commercial or financial relationships that could be construed as a potential conflict of interest. The handling editor declared a past co–authorship with author JH.
